# Supplementary material for: An integrative examination of psychological distress and its nutritional and visual correlates among young adults in Ghana
Source: PLoS One. 2025 Nov 17;20(11):e0329869. doi: 10.1371/journal.pone.0329869 (PMC12622780; doi:10.1371/journal.pone.0329869)
Supplement: Attachment — Submitted filename: Response to reviewers comments_IOD_10122025.pdf [file pone.0329869.s003.pdf]

PONE-D-25-36529: An integrative examination of psychological distress and its nutritional and visual correlates among young adults in Ghana

Dear Dr. Fagundo-Rivera,

We sincerely appreciate the opportunity to resubmit our manuscript titled *"An Integrative Examination of Psychological Distress and Its Nutritional and Visual Correlates Among Young Adults in Ghana."*

We are grateful for the reviewers' thoughtful and constructive feedback, which has substantially improved the quality and clarity of our work. All comments have been carefully addressed, and our detailed, point-by-point responses are provided below. Reviewer comments appear in *regular font*, while our responses are presented in bold. All corresponding revisions have been incorporated into the manuscript and are visible through *track changes*.

In addition, we have taken this opportunity to correct minor issues throughout the manuscript, also indicated in track changes.

The following files have been uploaded with this resubmission:

- **Response to Reviewers' Comments:** A detailed rebuttal letter addressing each reviewer's point.
- **Manuscript:** A clean, unmarked version of the revised paper.
- **Marked Manuscript with Track Changes:** A version highlighting all revisions made to the original submission.

We thank you once again for your time and consideration, and we hope that the revised version meets the journal's standards for publication.

Sincerely,
Dr. Isaiah Duah

on behalf of all co-authors

Reviewer #1:

We would like to thank the authors for their work on "An integrative examination of psychological distress and its nutritional and visual correlates among young adults in Ghana." The manuscript is well-structured and the topic is certainly of interest.

However, after reading the paper, we feel that the study unfortunately does not present new findings that would warrant publication. In its current form, we do not consider the manuscript suitable for publication.

In addition, the discussion section seems to be missing some important points. For example, the study shows no correlation between psychological distress and physical activity or biographical

factors, which stands in contrast to a number of previous studies reporting the opposite. This discrepancy should be addressed in detail to strengthen the discussion.

We appreciate the effort that went into this work and encourage the authors to further develop these aspects in future studies.

We sincerely thank the reviewers for their thoughtful feedback and for recognizing the interest and structure of our manuscript. We appreciate the opportunity to clarify the contributions and significance of our work.

While previous studies have explored psychological distress among young adults, few have comprehensively examined the nutritional, visual, and lifestyle correlates in the Ghanaian context using a multimodal approach. Our study is novel in combining self-reports and clinician assessments to capture both subjective and objective indicators of psychological distress. Importantly, we identify dietary intake of lutein and zeaxanthin, alcohol consumption, swimming activity, and visual function as significant correlates, providing evidence of modifiable factors that have not been previously explored in this population. These findings extend existing knowledge by linking nutritional, visual, and behavioral health with mental well-being, highlighting potential biological and lifestyle pathways for intervention.

We acknowledge that our study did not find significant correlations between psychological distress and other forms of physical activity or biographical factors, which contrasts with some prior research. Interestingly, swimming was associated with higher psychological distress in our sample, suggesting that individuals who swim may do so in response to stress or as part of coping behaviors rather than as a protective activity. This highlights the complex and potentially bidirectional relationships between lifestyle behaviors and mental health, warranting further investigation. The observed discrepancies in other physical activity outcomes may reflect contextual and population-specific differences, such as lifestyle patterns, dietary practices, and educational stressors unique to Ghanaian young adults. Highlighting these differences contributes to a more nuanced understanding of psychological distress across diverse populations and underscores the importance of localized research rather than assuming findings from other contexts are universally applicable.

The broader implications of our study are significant. By identifying modifiable nutritional, visual, and lifestyle risk factors, our findings inform potential multidisciplinary interventions, including diet-based strategies, vision correction programs, behavioral recommendations to reduce alcohol consumption, and consideration of activity-specific mental health risks. These interventions could meaningfully influence public health policies and mental health promotion strategies in emerging adults in Ghana and similar settings. Furthermore, the study lays the groundwork for future longitudinal research, including intervention trials assessing lutein and zeaxanthin supplementation, vision correction, and targeted lifestyle modifications as protective factors for psychological distress.

In conclusion, we respectfully contend that our study provides novel insights, intellectual merit, and practical relevance by addressing gaps in the literature, focusing on a previously

underexplored population, and identifying actionable factors for intervention. We are confident that, with these clarifications, the manuscript makes a meaningful contribution to the field of mental health research in young adults.

Reviewer #2:

This cross-sectional study of 301 Ghanaian young adults reports a 23.3% prevalence of psychological distress (K10 $\geq$ 20) and identifies associations with lower self-reported lutein/zeaxanthin intake, less eyeglass use, alcohol use, and higher (better) NEI-VFQ-25 scores being protective. The dataset is valuable, but the current analyses and presentation over-interpret some associations without adequate sensitivity checks, model diagnostics, or cautious language.

We sincerely appreciate your rigorous appraisal, which has guided us to reanalyze our data and interpret the findings with greater caution. Your insightful feedback enabled us to clearly delineate associations rather than implying causation, thereby enhancing the scientific rigor and credibility of our study.

Comments:
1. The causal language throughout the manuscript should be tempered. Given the cross-sectional design, I recommend reframing causal claims as associations and present intervention ideas as hypotheses for future trials.

We appreciate the reviewer's insightful comment. In accordance with this recommendation, we have carefully reviewed the manuscript and tempered any causal language to better reflect the cross-sectional nature of our study. All causal expressions have been reframed as associations, and intervention-related statements are now clearly presented as hypotheses for future research and experimental trials. These revisions ensure that our interpretations remain appropriately cautious and aligned with the study design.

2. I suggest re-analysing K10 without sole reliance on dichotomization. Presenting K10 as a continuous and/or ordinal outcome in sensitivity analyses would confirm robustness of findings.

We thank the reviewer for this constructive suggestion. In response, we re-analyzed the Kessler Psychological Distress Scale (K10) as an ordinal outcome variable with four categories: *no, mild, moderate,* and *severe psychological distress*. This approach avoids sole reliance on dichotomization and allows for a more nuanced interpretation of the data. The ordinal classification was applied consistently across all analyses, and Tables 1–3 have been updated to reflect results based on this ordinal outcome throughout. The ordinal logistic regression yielded findings that were directionally consistent with those from the dichotomized analyses, confirming the robustness and stability of our results. Marked manuscript, pages 9-18, lines 194-286.

"Results

Psychological distress is linked to ethnicity, alcohol use, and eyewear factors but not to physiological or physical activity measures

Psychological distress was unevenly distributed in the sample, with most participants reporting no distress (76.7%), followed by mild (14.6%), severe (5.6%), and moderate distress (3.0%). Biographical characteristics such as age and sex showed no notable differences in distress levels, whereas ethnicity demonstrated a small but significant distribution effect ($p = 0.013$). Health measures, including blood pressure and medication use, were similarly evenly distributed across distress categories ($p > 0.05$). Lifestyle factors largely showed uniform distributions, though alcohol consumption exhibited a significant difference ($p = 0.038$), indicating higher distress among some drinkers. Use of eyewear was not significantly associated with distress levels ($p = 0.176$), although the form of eyewear showed a significant difference ($p = 0.016$), while the purpose and frequency of use did not significantly affect distress distribution ($p > 0.05$). Physical activity patterns, including type, frequency, and total activity scores, were not significantly associated with differences in distress distribution ($p > 0.05$) see Table 1.

Table 1: Biographical, health, lifestyle, and physical activity correlates of psychological distress among young adults from Ghana

**Variable**	**Psychological distress % (n)**				**Test-statistic**	**Effect size**	**p-value**
Level of psychological distress	No distress	Mild distress	Moderate distress	Severe distress			
Kessler scale	(K10 ≤ 19)	(K10 20–24)	(K10 25–29)	(K10 ≥ 30)			
Prevalence of PSD	76. 7 (231)	14.6 (44)	3.0 (9)	5.6 (17)			
**Biographical measures**							
Age, years (mean ± SE)	21.16 ± 0.13	20.98 ± 0.31	20.56 ± 0.58	21.06 ± 0.46	F = 0.378	η^2 = 0.004	p = 0.769
Sex							
Male	41.2 (124)	6.6 (20)	1.7 (5)	2.3 (7)	χ^2 = 1.858	V = 0.079	p = 0.603
Female	35.5 (107)	8.0 (24)	1.3 (4)	3.3 (10)			
Ethnicity							
Akan	61.1 (184)	10.6 (32)	2.7 (8)	4.3 (13)	χ^2 = 29.619	V = 0.181	p = 0.013
Ga	2.7 (8)	0.7 (2)	0.0 (0)	0.0 (0)			
Ewe	7.0 (21)	0.7 (2)	0.3 (1)	0.3 (1)			
Northerners	4.0 (12)	1.7 (5)	0.0 (0)	0.0 (0)			
Guan	1.0 (3)	0.7 (2)	0.0 (0)	0.0 (0)			
Others	1.0 (3)	0.3 (1)	0.0 (0)	1.0 (3)			
**Health status measures**							
Blood pressure, mm Hg (mean ± SE)							

Diastolic	70.33 ± 0.58	69.48 ± 1.26	64.89 ± 3.09	70.29 ± 2.07	F = 1.206	η^2 = 0.012	p = 0.308
Systolic	107.82 ± 0.90	106.11±2.07	100.67 ± 2.91	106.76 ±2.78	F = 0.692	η^2 = 0.010	p = 0.406
Medication Use							
No	72.1 (217)	13.6 (41)	3.0 (9)	4.7 (14)	χ^2 = 4.087	V = 0.117	p = 0.252
Yes	4.7 (14)	1.0 (3)	0.0 (0)	1.0 (3)			
**Lifestyle measures**							
Secondhand smoke							
Not Exposed	71.1 (214)	13.0 (39)	2.7 (8)	4.7 (14)	χ^2 = 2.711	V = 0.095	p = 0.438
Exposed	5.6 (17)	1.7 (5)	0.3 (1)	1.0 (3)			
Alcohol consumption							
None (never drink)	69.8 (210)	12.6 (38)	2.7 (8)	3.7 (11)	χ^2 = 13.350	V = 0.149	p = 0.038
Special occasion	5.3 (16)	2.0 (6)	0.3 (1)	1.7 (5)			
1-2 times a month	1.7 (5)	0.0 (0)	0.0 (0)	0.3 (1)			
Volume of alcohol consumption							
1 units per week	76.5 (13)	5.9 (1)	0.0 (0)	11.8 (2)	χ^2 = 4.958	V = 0.540	p = 0.084
6-10 units per week	0.0 (0)	0.0 (0.0)	0.0 (0)	5.9 (1)			
Light exposure							
Little (<50% of the day outside)	34.2 (103)	4.3 (13)	0.7 (2)	3.0 (9)	χ^2 = 6.006	V = 0.141	p = 0.422
Some (50% outside and 50% inside)	32.9 (99)	7.6 (23)	1.7 (5)	2.0 (6)			
Most (Spends >50% of the time outside)	9.6 (29)	2.7 (8)	0.7 (2)	0.7 (2)			
Use of eye wear							
Yes	26.2 (79)	7.0 (21)	1.7 (5)	2.7 (8)	χ^2 = 4.950	V = 0.128	p = 0.176
No	50.5 (152)	7.6 (23)	1.3 (4)	3.0 (9)			
Form of eye wear							
None	57.5 (173)	8.0 (24)	1.3 (4)	3.7 (11)	χ^2 = 15.631	V = 0.161	p = 0.016
Spectacles	19.3 (58)	6.3 (19)	1.7 (5)	2.0 (6)			
Contact lens	0.0 (0)	0.3 (1)	0.0 (0)	0.0 (0)			
Purpose of eye wear							
Distant activity	37.1 (33)	10.1 (9)	2.2 (2)	3.4 (3)	χ^2 = 15.631	V = 0.117	p = 0.873
Near activity	7.9 (7)	1.1 (1)	1.1 (1)	1.1 (1)			
Both	21.3 (19)	10.1 (9)	2.2 (2)	2.2 (2)			

Frequency of use of eyewear							
Occasionally	10.1 (9)	3.4 (3)	1.1 (1)	0.0 (0)	χ^2 = 6.612	V = 0.193	p = 0.358
Part-time	24.7 (22)	11.2 (10)	0.0 (0)	2.2 (2)			
Full-time	31.5 (28)	6.7 (6)	4.5 (4)	4.5 (4)			
**Physical activity measures**							
Type of physical activity							
Walking, yes	76.7 (231)	14.6 (44)	3.0 (9)	5.6 (17)	nd	nd	nd
Running, yes	10.3 (31)	1.3 (4)	0.3 (1)	0.0 (0)	χ^2 = 3.127	V = 0.102	p = 0.372
Swimming, yes	1.7 (5)	1.0 (3)	0.0 (0)	0.3 (1)	χ^2 = 3.533	V = 0.108	p = 0.316
Cycling, yes	1.7 (5)	0.0 (0)	0.0 (0)	0.3 (1)	χ^2 = 2.429	V = 0.090	p = 0.488
Gym-based workout, yes	7.3 (22)	2.7 (8)	0.0 (0)	0.0 (0)	χ^2 = 6.238	V = 0.144	p = 0.101
Team-based sports, yes	5.6 (17)	0.3 (1)	0.0 (0)	0.3 (1)	χ^2 = 2.254	V = 0.087	p = 0.521
Duration of physical activity in mins							
Walking							
Running	11.30 ± 3.53	2.23 ± 1.33	1.56 ± 1.56	0.0 ± 0.0	F = 0.760	η^2 = 0.008	p = 0.517
Swimming‡	149.71	156.9	146.5	155.59	H = 3.719	ε^2 = 0.002	p = 0.293
Cycling	1.21 ± 0.58	0.0 ± 0.0	0.0 ± 0.0	0.29 ± 0.29	F = 0.399	η^2 = 0.004	p = 0.754
Gym-based workout‡	149.79	163.28	135.5	143.82	H = 4.751	ε^2 = 0.006	p = 0.191
Team-based sports‡	10	2		8	H = 4.151	ε^2 = 0.077	p = 0.125
Total physical activity scores	380.42 ± 25.00	416.66 ± 75.04	473.11 ± 148.18	380.18 ± 137.74	F = 0.226	η^2 = 0.002	p = 0.878

Note: *n*, frequency (may not equal 301 for all variables); %, percentage frequency; SE, standard error. Normality of the data was assessed using the Kolmogorov–Smirnov test. Where appropriate, group differences were analyzed using independent one-way ANOVA with *F*-statistic and Eta-squared (η^2) effect size, Kruskal–Wallis one-way ANOVA with Kruskal–Wallis statistic (*H*) and epsilon-squared (ε^2) effect size with ‡ mean rank, or chi-squared analysis with Pearson chi-square statistic (χ^2) and Cramer's *V* (V) effect size. Statistical significance was set at $p \leq 0.05$.

Psychological distress is linked to lower carotenoid intake and poorer subjective visual function but not objective physiological or cognitive measures

Table 2 presents the relationship between psychological distress and a range of dietary, anthropometric, visual, and cognitive measures among participants (Table 2). Psychological distress was significantly associated with lower dietary intake of lutein, zeaxanthin, and their combined intake (all $p < 0.05$; **Fig. 1**). Participants with psychological distress had substantially lower consumption of these carotenoids compared with those without distress. However, when levels of distress severity (mild, moderate, severe) were examined, this difference was annulled, suggesting that the relationship between carotenoid intake and distress was more pronounced in the presence versus absence of distress, but did not vary significantly across the different severity levels (p>0.005, for all). Vitamin or supplement use was not significantly related to distress (p = 0.338), indicating that supplement habits were not associated with psychological health. Body composition indices, including fat mass, visceral fat, lean mass, muscle mass, and BMI, showed no significant variation among the distress groups (all $p > 0.05$). Participants with severe psychological distress had somewhat higher BMI and body fat values on average, but these differences were not statistically significant (p > 0.05). When BMI was categorized into healthy, underweight, overweight, and obese, no significant differences were found (p = 0.125), further suggesting that body composition did not play a major role in explaining psychological distress. Visual performance measures, including best-corrected visual acuity, contrast sensitivity, and macular pigment optical density (measured at 0.5° and 1°), showed no significant differences across distress levels (all $p > 0.05$). However, subjective visual function (VFQ-25 scores) differed significantly between individuals with and without psychological distress (p = 0.001), and this difference persisted across levels of distress severity (p = 0.002). Participants with low distress reported the highest perceived visual function, whereas those with moderate distress reported the lowest, suggesting that perceived rather than objective visual quality was more closely linked to emotional well-being. Neuropsychological measures showed a similar pattern. Global cognitive function, assessed by the Mini-Mental State Examination (MMSE), approached significance (p = 0.056), with participants experiencing mild distress scoring slightly higher than others. Verbal fluency measures (F-A-S and Animal Fluency) did not differ significantly ($p > 0.05$), indicating stable cognitive performance across distress levels (**Table 2**).

Table 2. Psychological distress in relation to dietary intake, body composition, visual performance, and cognitive measures among young adults from Ghana

**Variable**	**Psychological distress**				**Test-statistic**	**Effect size**	**p-value**
Level of psychological distress	No distress	Mild distress	Moderate distress	Severe distress			
	Mean ± SE	Mean ± SE	Mean ± SE	Mean ± SE			
**Dietary measures**							
Lutein (L), *mg/d*	4.47±0.74	3.02 ± 1.32	0.66 ± 0.27	3.01 ± 1.81	F = 0.629	η^2 = 0.006	p = 0.597
Zeaxanthin (Z), *mg/d*	0.37 ± 0.17	0.20 ± 0.04	0.11 ± 0.03	0.13 ± 0.04	F = 0.147	η^2 = 0.001	p = 0.932

L + Z, *mg/d*	4.84 ± 0.82	3.22 ± 1.32	0.92 ± 0.31	3.14 ± 1.80	F = 0.600	η^2 = 0.001	p = 0.615
Regular vitamins or supplement							
Vitamins or supplement use	8.6 (26)	2.7 (8)	0.3 (1)	1.3 (4)	χ^2 = 3.370	V = 0.106	p = 0.338
No vitamins or supplement	68.1 (205)	12.0 (36)	2.7 (8)	4.3 (13)			
**Anthropometric measures**							
Fat Mass or Body Fat (FM)	26.25 ± 1.64	27.92 ± 1.76	22.31 ± 3.33	30.25 ± 3.84	F = 0.324	η^2 = 0.003	p = 0.808
Fat Mass Index in kg/m^2 (FMI)	9.61 ± 0.68	10.31 ± 0.72	7.75 ± 1.31	11.04 ± 1.44	F = 0.308	η^2 = 0.003	p = 0.820
Visceral Fat (FT)	4.38 ± 0.15	4.91 ± 0.15	4.33 ± 0.65	4.71 ± 0.57	F = 0.657	η^2 = 0.007	p =0.579
Lean Mass (LM)	37.70 ± 1.74	38.43 ± 2.21	43.94 ± 5.02	42.13 ± 3.78	F = 0.348	η^2 = 0.004	p = 0.790
Lean Mass Index in kg/m^2 (LMI)	13.04 ± 0.66	13.51 ± 0.63	14.61 ± 1.39	15.03 ± 1.26	F = 0.341	η^2 = 0.003	p = 0.796
Muscle Mass (MM)‡	153.07	132.44	148.39	146.38	H = 2.139	ε^2 = 0.002	p = 0.544
Waist circumference‡	147.67	152.36	158.5	179.76	H = 1.739	ε^2 = 0.004	p = 0.628
BMI in kg/m^2	23.77 ± 0.86	24.50 ± 0.94	22.41 ±0.80	26.0 ± 2.03	F = 0.280	η^2 = 0.003	p = 0.840
BMI in kg/m$^{2(Cat)}$							
Healthy	50.0 (149)	8.1 (24)	2.7 (8)	3.4 (10)	χ^2 = 13.924	V = 0.125	p = 0.125
Underweight	6.7 (20)	1.3 (4)	0.0 (0.0)	0.0 (0.0)			
Overweight	16.4 (49)	3.0 (9)	0.3 (1)	1.7 (5)			
Obese	3.4 (10)	2.3 (7)	0.0 (0.0)	0.7 (2)			
**Visual performance measures**							
Best corrected visual acuity							
Oculus dexter	-0.20 ± 0.01	-0.21 ± 0.01	-0.22 ± 0.03	-0.22 ± 0.03	F = 0.432	η^2 = 0.004	p = 0.730
Oculus sinister	-0.20 ± 0.01	-0.21 ± 0.01	-0.21 ± 0.01	-0.19 ± 0.02	F = 0.112	η^2 = 0.001	p = 0.593
Contrast sensitivity function							
Oculus dexter	1.61 ± 0.002	1.61 ± 0.004	1.62 ± 0.008	1.62 ± 0.006	F = 0.337	η^2 = 0.003	p = 0.799

Oculus sinister	1.61 ± 0.004	1.61 ± 0.004	1.62 ± 0.008	1.62 ± 0.007	F = 0.075	η^2 = 0.001	p = 0.973
Macular pigment optical density							
MPOD at 1°	0.35 ± 0.01	0.32 ± 0.03	0.33 ± 0.03	0.32 ± 0.03	F = 0.516	η^2 = 0.005	p = 0.672
MPOD at 0.5°	0.37 ± 0.01	0.35 ± 0.03	0.38 ± 0.03	0.33 ± 0.04	F = 0.576	η^2 = 0.006	p = 0.631
Subjective visual function							
NEI- VFQ-25‡	160.6	130.56	85.2	107.97	H = 14.481	ε^2 = 0.039	p =0.002
**Neuropsychological function**							
MMSE‡	147.55	180.98	128.83	132.09	H = 7.576	ε^2 = 0.015	p = 0.056
F-A-S	43.59 ± 0.73	43.64 ± 1.89	40.33 ± 2.92	40.53 ± 2.28	F = 0.625	η^2 = 0.006	p = 0.599
Animal Fluency	16.18 ± 0.25	17.48 ± 0.60	16.44 ± 1.06	15.82 ± 1.12	F = 1.150	η^2 = 0.015	p = 0.212

Note: *n*, frequency (may not equal 301 for all variables); %, percentage frequency; SE, standard error. Normality of the data was assessed using the Kolmogorov–Smirnov test. Where appropriate, group differences were analyzed using independent one-way ANOVA with *F*-statistic and Eta-squared (η^2) effect size, Kruskal–Wallis one-way ANOVA with Kruskal–Wallis statistic (*H*) and epsilon-squared (ε^2) effect size with ‡ mean rank, or chi-squared analysis with Pearson chi-square statistic (χ^2) and Cramer's *V* (V) effect size. Statistical significance was set at $p \leq 0.05$. MMSE, Mini-Mental State Examination; F-A-S, phonemic verbal fluency test; NEI-VFQ-25, National Eye Institute Visual Function Questionnaire–25; MPOD, macular pigment optical density.

Factors influencing the severity of psychological distress

The results of the ordinal logistic regression analyses suggest that several lifestyles, dietary, and visual performance factors are associated with the severity of psychological distress, while many biographical, anthropometric, and physical activity measures were not significant (see Table 3). In the bivariate analyses, alcohol consumption on occasion, use of protective eyewear, spectacles, swimming duration, and VFQ-25 scores were significantly associated with severity of psychological distress. In the multivariate model, occasional alcohol consumption (AOR = 4.10, 95% CI: 1.63–10.12, p = 0.003) was associated with a significant increase in the odds of higher severity of psychological distress. Spending more than 50% of the day outside (AOR = 2.40, 95% CI: 0.97–5.81, p = 0.054) showed a trend toward increased odds, though it was marginally non-significant. Longer swimming duration (AOR = 1.04, 95% CI: 1.01–1.09, p = 0.037) was also associated with a slight increase in odds of higher severity. Conversely, higher VFQ-25 scores (AOR = 0.95, 95% CI: 0.91–0.99, p = 0.008) were associated with a reduction in the odds of experiencing more severe psychological distress, suggesting better visual function may be protective. Other variables, such as ethnicity, protective eyewear, BMI category, and dietary intake of lutein and zeaxanthin, were not statistically significant after adjustment (p>0.05), suggesting their effects may be confounded by other covariates. Overall, these findings highlight that certain behavioral and visual function factors can increase or reduce the odds of higher severity of

psychological distress, while many demographic, health, and anthropometric measures appear less influential in this population (see **Table 3**).

Table 3: Bivariate and multiple ordinal regression analysis of factors associated to severity of psychological distress

Variable	Bivariate ordinal logistic regression			Multivariate ordinal regression		
	COR	95% CI (Lower, Upper)	p-value	AOR	95% CI (Lower, Upper)	p-value
**Biographical measures**						
Age, years	0.94	0.82,1.08	0.408			
Sex						
Male	Ref					
Female	1.37	0.80, 2.34	0.243			
Ethnicity						
Akan	Ref					
Ewe	0.49	0.11, 1.50	0.269			
Ga	1.23	0.27, 4.25	0.758			
Northerners	1.24	0.39, 3.36	0.690			
Guan /Others	3.79	1.17, 11.71	**0.022**	3.57	0.69, 16.94	0.115
**Health status measures**						
Blood pressure , mm Hg						
Diastolic	0.98	0.95, 1.01	0.290			
Systolic	0.99	0.97, 1.01	0.219			
Medication Use						
Yes	1.57	0.58, 4.26	0.371			
No	Ref					
**Lifestyle measures**						
Exposure to secondhand smoke						

Exposed to secondhand smoke	1.91	0.82, 4.45	0.133	1.79	0.66,4.52	0.234
Not Exposed to secondhand smoking	Ref					
Alcohol consumption						
No alcohol	Ref					
Alcohol on occasion	2.99	1.33, 6.50	**0.007**	4.10	1.63, 10.12	**0.003**
Alcohol use once or twice a month	0.88	0.04, 5.82	0.909			
Light exposure						
Spends <50% of the day outside	Ref					
50% outside and 50% inside	1.40	0.78, 2.54	0.263			
Spends >50% of the time outside	1.67	0.74, 3.66	0.206	2.40	0.97,5.81	0.054
Protective eyewear						
Yes	1.80	1.05, 3.09	**0.032**	1.11	0.50,2.39	0.797
No	Ref					
Form of eye wear						
None						
Spectacles	2.22	1.27,3.85	**0.005**	1.28	0.55, 3.00	0.567
Contact lenses	7.99	0.29,222.97	0.164	2.56	0.07, 98.02	0.582
Purpose of eye wear						
Distant	Ref					
Near	1.15	0.22, 4.78	0.855			
Both	1.52	0.60,3.82	0.375			
Frequency of use of eye wear						
Occasionally	Ref					
Part-time	1.20	0.33, 5.02	0.792			

Full-time	1.32	0.37, 5.47	0.676			
**Physical activity measures**						
Routine physical activity						
Running, yes	0.48	0.18, 1.28	0.143	1.51	0.23,8.62	0.649
Swimming, yes	2.44	0.68, 8.73	0.170	0.01	0.00,0.55	0.085
Cycling, yes	0.78	0.09, 6.98	0.821			
Gym-based workout, yes	1.06	0.46, 2.47	0.886			
Duration of physical activity						
Walking	1.00	1.00,1.00	0.526			
Running	0.98	0.95,1.01	0.109	0.97	0.91,1.00	0.142
Swimming	1.01	1.00,1.02	**0.008**	1.04	1.01,1.09	**0.037**
Cycling	0.95	0.83,1.09	0.463			
Gym-based workout	1.00	1.00,1.00	0.724			
Team-based sports	1.01	0.99,1.02	0.386			
Total physical activity scores	1.00	1.00, 1.00	0.571			
**Dietary measures**						
Lutein, *mg/d*	0.98	0.95, 1.01	0.226			
Zeaxanthin, *mg/d*	0.36	0.08, 1.66	0.190	0.32	0.05, 0.93	0.172
Lutein + Zeaxanthin, *mg/d*	0.98	0.95, 1.01	0.224			
Regular vitamins or supplement						
Vitamins or supplement use	1.80	0.88, 3.69	0.108	1.86	0.82,4.05	0.127
No vitamins or supplement	Ref					
**Anthropometric measures**						
Fat Mass or Body Fat (FM)	1.00	0.99,1.01	0.621			
Fat Mass Index in kg/m^2 (FMI)	1.01	0.98, 1.03	0.674			
Visceral Fat (FT)	1.06	0.96,1.18	0.255			

Lean Mass (LM)	1.01	0.99,1.02	0.428			
Lean Mass Index in kg/m^2 (LMI)	1.03	0.97,1.09	0.359			
Muscle Mass (MM)	1.01	0.98, 1.03	0.623			
Waist circumference	1.02	1.00,1.05	0.099	1.02	0.98, 1.06	0.312
BMI in kg/m^2	1.01	0.99,1.03	0.592			
BMI in kg/m$^{2\ Cat}$						
Healthy	0.65	0.18,1.82	0.457			
Underweight	Ref					
Overweight	1.09	0.55,2.09	0.800			
Obese	2.70	1.06,6.57	**0.032**	0.80	0.18,3.26	0.763
**Visual performance measures**						
Best corrected visual acuity (BCVA)						
Oculus dexter	0.76	0.04,12.86	0.846			
Oculus sinister	0.72	0.08,6.95	0.779			
Contrast sensitivity function						
Oculus dexter	0.73	0.00, 6541.35	0.947			
Oculus sinister	1.95	0.01,336.22	0.798			
Macular pigment optical density (MPOD)						
MPOD at 1°	0.35	0.06, 2.12	0.251			
MPOD at 0.5°	0.39	0.07, 2.12	0.274			
VFQ-25	0.94	0.91, 0.97	**<0.001**	0.95	0.91, 0.99	**0.008**
**Neuropsychological function**						
MMSE	0.94	0.80, 1.10	0.408			
FAS	0.99	0.97, 1.01	0.383			
Animal fluency	1.04	0.97, 1.12	0.220			

Dependent Variable, Psychological Distress on ordinal scale. OR, Odds ratio; CI, Confidence Interval; AOR, Adjusted Odds Ratio; VFQ-25, The National Eye Institute 25-Item Visual Function Questionnaire; MMSE, Mini-Mental State Examination; FAS, F-A-S phonemic verbal fluency test. Bivariate ordinal logistic regression at Wald test ≤ 0.2 considered for inclusion in the multivariate model; Statistical significance set at $p \leq 0.05$"

3. I suggest clarifying an apparent inconsistency regarding lutein/zeaxanthin. When participants were categorized into two groups (with vs without physiological distress; Table 2), lutein/zeaxanthin showed a significant contribution, yet the logistic regression (Table 3) returned a non-significant effect. The manuscript currently implies diet is an important predictor; I recommend presenting the unadjusted and adjusted results side-by-side and explicitly discussing reasons for the discrepancy.

We appreciate the reviewer's insightful observation regarding the apparent inconsistency in the association between dietary lutein/zeaxanthin and psychological distress. In response, we have clarified this issue in both the Results and Discussion sections. The significant difference observed in Table 2 reflects the bivariate (unadjusted) association, whereas the multivariable (adjusted) ordinal logistic regression model in Table 3 accounts for potential confounders such as age, sex, alcohol consumption, vision status, and outdoor exposure. When these covariates were included, the independent effect of lutein/zeaxanthin intake attenuated and lost statistical significance. To enhance transparency, we have now presented both unadjusted and adjusted estimates side by side in Table 3 and have explicitly discussed the potential reasons for this attenuation, likely due to shared variance with correlated behavioral and health factors. This clarification underscores that while lutein/zeaxanthin intake shows a noteworthy crude association, its independent predictive effect diminishes after controlling for confounders, suggesting an indirect or context-dependent relationship rather than a direct causal influence. Marked manuscript, page 20, lines 320-334.

"Participants experiencing psychological distress reported lower dietary intakes of lutein and zeaxanthin (Figure 1). These xanthophyll carotenoids, primarily derived from fruits and vegetables, accumulate in the retina and brain and are known to support visual and cognitive functioning [14, 15, 20]. Previous studies have linked reduced levels of these nutrients to increased psychological stress [12]. Lutein and zeaxanthin are carotenoids with antioxidant and anti-inflammatory properties that support both retinal and neural function [59]. Deficiencies in these nutrients may impair visual processing and increase oxidative stress in the brain, potentially contributing to mood disturbances and emotional dysregulation [12]. However, when psychological distress is assessed according to severity rather than a simple presence/absence, the differences in lutein and zeaxanthin intake are attenuated. This attenuation may reflect the multifactorial nature of more severe distress, where dietary factors are only one of many contributors, alongside genetic predisposition, environmental stressors, and lifestyle behaviors (Table 2). Collectively, these findings highlight the potential value of nutrition-based interventions and educational programs aimed at promoting both visual and mental health among young adults [12]."

4. I suggest improving alcohol measurement reporting. “Alcohol on special occasions” is ambiguous—please provide frequency/volume distributions and test for dose–response if data permit; otherwise acknowledge this limitation.

We thank the reviewer for this suggestion. Alcohol consumption in our study was assessed based on self-reported intake “on special occasions,” with participants indicating both the frequency of such occasions (e.g., monthly, weekly) and typical volume consumed per occasion, categorized as 1 unit, 2–5 units, or 6–10 units. Due to the relatively small number of participants reporting regular alcohol use and the variability across categories, we were unable to conduct formal dose–response analyses. We have added these details to the Methods section and acknowledged this limitation in the Discussion, emphasizing the need for future studies to collect more detailed frequency and volume data to better examine potential dose–response relationships with psychological distress. Marked manuscript, Discussion, page 24, lines 410-414.

“Additionally, due to the relatively small number of participants reporting regular alcohol use and the variability across consumption categories, we were unable to conduct formal dose–response analyses. Future studies should aim to recruit larger samples of participants who consume alcohol frequently to allow for more robust dose–response investigations and clearer understanding of potential associations with psychological distress.”

5. I recommend that the authors discuss subjective versus objective vision findings more explicitly. The null results for BCVA, contrast sensitivity, and MPOD alongside a strong NEI-VFQ-25 association may reflect perceived vision or reporting bias; avoid implying spectacle provision will necessarily reduce distress without experimental evidence.

We thank the reviewer for this insightful comment. We have now more explicitly discussed the distinction between subjective and objective vision measures. While our study found no significant associations between psychological distress and objective measures such as BCVA, contrast sensitivity, or MPOD, we observed a strong association with self-reported visual function on the NEI-VFQ-25. This suggests that perceived vision difficulties, rather than measured visual deficits, may be more closely linked to psychological distress, potentially reflecting reporting bias or the psychosocial impact of vision limitations. We have also tempered any language implying that spectacle provision would necessarily reduce distress, emphasizing that experimental studies would be required to establish causality. Marked manuscript, Discussion, page 22, lines 364-377.

“Further, participants with better self-reported visual function, as indicated by higher NEI-VFQ-25 scores, were less likely to experience psychological distress. This finding aligns with previous studies showing that self-perceived vision impairment is associated with higher

emotional distress and depressive symptoms [24, 66]. Interestingly, this association was observed for subjective, rather than objective, visual measures. One possible explanation is that self-reported vision reflects not only measurable visual acuity but also the functional and psychosocial impact of vision on daily life, including the ability to engage in work, study, or social activities [24, 66, 67]. In contrast, objective measures may not fully capture the practical limitations or frustrations individuals experience, which could influence psychological well-being [24]. Since participants without mental distress were more likely to wear spectacles, early detection and correction of vision problems could represent an overlooked, yet biologically plausible, avenue for preventing or alleviating psychological distress among young adults (Table 2). Accordingly, we recommend routine eye care screening to detect early vision difficulties that may contribute to psychological distress in this population."

6. In Methods, the K10 response options are described with a duplicated phrase ("most of the time" appears twice)

We have deleted for consistency.

Reviewer #3:

I read with great interest this manuscript on the psychological distress and its nutritional and visual correlates amoung young adults in Ghana. Please find my comments below.

Thank you for the opportunity to have our manuscript reviewed. We sincerely appreciate the critical feedback, which has helped improve the quality of our manuscript.

Line 87 - 92: A brief summary of the study results and future directions is included at the end of the introduction. However, this content would be more appropriate at the end of the discussion and conclusion section. The introduction should instead focus on presenting the aim and significance of the study.

We have removed the summary of the findings and replaced it with a discussion of the study's significance. Please refer to the Introduction, page 5, lines 102–108.

"This study therefore aimed to comprehensively assess psychological distress among young adults in Ghana, with a focus on nutritional and visual correlates. A multimodal approach was employed, combining self-reported questionnaires with clinician-administered assessments. The significance of this research lies in its potential to address critical gaps in mental health knowledge in Ghana, providing evidence-based insights into modifiable lifestyle factors, informing the design of early detection programs, and guiding policy initiatives to reduce the burden of psychological distress and enhance the overall quality of life among young adults."

Line 96: Replace "and" with "to".

We have rephrased the entire paragraph to incorporate this changes.

Line 160: The SPSS stands for Statistical Product (not package) and Service Solutions. However, it is uncommon to use the full name and not many people know it. I suggest referring to it as SPSS to avoid any confusion.

Thank you very much for pointing. We have incorporated the corrections as suggested as seen in Methods, page 8, lines 175.

"…Statistical Product and Service Solution version 25…"

Line 161: In the methods section Shapiro-Wilk is mentioned as the statistical test utilized for normality testing. However, at line 192 Kolmogorov-Smirnov seems to be utilized.

We have revised the manuscript to explicitly mention the Kolmogorov-Smirnov test in the text and under the relevant tables, as shown below and in the revised manuscript (Methods, page 8, line 177).

"…Kolmogorov-Smirnov statistics …"

Line 315: I highly recommend removing the last sentence: "potentially contribute to reduce suicides among young adult population from Ghana". Although correcting vision problems, high lutein and zeaxanthin intake, and decreased alcohol consumption may have ameliorating effects on psychological distress, due to the cross-sectional design of the study conclusions must be made with caution and limitations of study design in mind.

Thank you for pointing this out. We have revised the conclusion to incorporate your suggestions, as reflected below and in the revised manuscript (Conclusion, pages 19–20, lines 347–355).

"Among young adults, one in five was found to experience psychological distress. Most of these individuals reported low intakes of xanthophyll carotenoids (lutein and zeaxanthin) and lower use of eyewear. Alcohol consumption and better self-reported vision demonstrated opposite associations, with alcohol increasing the likelihood of psychological distress and good vision appearing protective. This high burden underscores the need for institutionalized mental health screening programs to detect early symptoms and provide timely coping interventions. Additionally, integrating eye care services to identify and correct vision problems, along with dietary education to optimize lutein and zeaxanthin intake while reducing alcohol consumption, may help ameliorate the risk of psychological distress among young adults in Ghana."

Reviewer #4:

This is a well-conducted and clearly written study that adds valuable insights to the literature. The revisions needed are minor and relate mainly to enhancing clarity. I encourage the authors to expand the discussion of limitations, particularly regarding sample representativeness and

generalizability, and to streamline some overlapping details in the methods and discussion sections. Strengthening the link between findings and practical implications will further improve the paper's impact. With these modest revisions, the manuscript will be well-positioned for publication.

Thank you very much for the thorough critical appraisal, which has significantly helped us improve the scientific merit and readability of our paper for greater impact. As suggested, we have meticulously expanded the discussion of the limitations and streamlined redundancies, as reflected below and in the revised manuscript (Discussion, pages 18–19, lines 314–336).

"Factors accounting for psychological distress among young adults in Ghana remain unclear. In this novel investigation, we provide comprehensive evidence of an increased burden of psychological distress among young adults. Taken together, this demonstrates the intellectual merit of this study in generating prevalence data, which are essential for contributing to systematic reviews and informing future longitudinal studies. We have also identified high-risk behaviors and highlighted modifiable factors that may help reduce this psychological burden. These findings have significant wider implications that may inform institutional and national intervention policies and pave the way for developing coping strategies for populations at high risk of psychological distress. Further, regarding sample representativeness, our study recruited *university students* ***using*** *systematic random sampling* ***. Universities provide a reasonably representative cross-section of young adults, encompassing diverse backgrounds in terms of age, sex, socioeconomic status, and educational attainment. While our sample reflects key demographic characteristics of the target population, some subgroups may still be underrepresented, which could limit the generalizability of our findings. Nevertheless, recruiting participants from multiple universities using a systematic approach enhances external validity, and our results likely provide meaningful insights applicable to broader young adult populations in Ghana and similar contexts. It is worth mentioning that our study has limitations. Assessment of lutein and zeaxanthin was based on dietary recall, which may not fully reflect true intake due to recall bias. However, this approach has been shown to provide robust estimates consistent with plasma profiling of carotenoids and is widely used in clinical and epidemiological studies [52]. Intimate partner relationships are common among young adults and may influence mental health. Although our study did not investigate this, future research assessing their impact could clarify some of the observed associations and further inform strategies to mitigate psychological distress [53]."***
